# The myosin and RhoGAP MYO9B influences osteocyte dendrite growth and responses to mechanical stimuli

**DOI:** 10.3389/fbioe.2023.1243303

**Published:** 2023-08-22

**Authors:** Beth S. Lee, Cynthia Murray, Jie Liu, Minji Kim, Min Sik Hwang, Tina Yueh, Myrna Mansour, Sana Qamar, Gunjan Agarwal, Do-Gyoon Kim

**Affiliations:** ^1^ Department of Physiology and Cell Biology, College of Medicine, The Ohio State University, Columbus, OH, United States; ^2^ Division of Orthodontics, College of Dentistry, The Ohio State University, Columbus, OH, United States; ^3^ Department of Orthodontics, Graduate School of Clinical Dentistry, Ewha Womans University, Seoul, Republic of Korea; ^4^ Department of Mechanical and Aerospace Engineering, College of Engineering, The Ohio State University, Columbus, OH, United States

**Keywords:** bone, osteocytes, osteoblasts, mechanosignaling, RhoA, RhoGAP

## Abstract

**Introduction:** Myosin IXB (MYO9B) is an unconventional myosin with RhoGAP activity and thus is a regulator of actin cytoskeletal organization. MYO9B was previously shown to be necessary for skeletal growth and health and to play a role in actin-based functions of both osteoblasts and osteoclasts. However, its role in responses to mechanical stimulation of bone cells has not yet been described. Therefore, experiments were undertaken to determine the role of MYO9B in bone cell responses to mechanical stress both *in vitro* and *in vivo*.

**Methods:** MYO9B expression was knocked down in osteoblast and osteocyte cell lines using RNA interference and the resulting cells were subjected to mechanical stresses including cyclic tensile strain, fluid shear stress, and plating on different substrates (no substrate vs. monomeric or polymerized collagen type I). Osteocytic cells were also subjected to MYO9B regulation through Slit-Robo signaling. Further, wild-type or *Myo9b* ^*−/−*^ mice were subjected to a regimen of whole-body vibration (WBV) and changes in bone quality were assessed by micro-CT.

**Results:** Unlike control cells, MYO9B-deficient osteoblastic cells subjected to uniaxial cyclic tensile strain were unable to orient their actin stress fibers perpendicular to the strain. Osteocytic cells in which MYO9B was knocked down exhibited elongated dendrites but were unable to respond normally to treatments that increase dendrite length such as fluid shear stress and Slit-Robo signaling. Osteocytic responses to mechanical stimuli were also found to be dependent on the polymerization state of collagen type I substrates. Wild-type mice responded to WBV with increased bone tissue mineral density values while *Myo9b* ^*−/−*^ mice responded with bone loss.

**Discussion:** These results demonstrate that MYO9B plays a key role in mechanical stress-induced responses of bone cells *in vitro* and *in vivo*.

## Introduction

Myosins are actin-based motor proteins that perform a wide variety of cellular activities facilitated by structural and functional heterogeneity in their tail domains. Thirty-five classes of myosins are present in eukaryotes, with twelve classes expressed by humans, including class IX (Odronitz and Kollmar 2007). Myosin IXB (MYO9B) is one of only two class IX members, which are unique among myosins due to the presence of a RhoGAP domain in their tails. RhoGAPs (Rho GTPase-activation proteins), along with RhoGEFs (Rho guanine nucleotide exchange factors), are regulators of the Rho superfamily of GTPases, and therefore are integral to regulating the dynamics of the actin cytoskeleton (Bos et al., 2007). Rho GTPases are inhibited by RhoGAPs due to their acceleration of GTP hydrolysis that drives the GTPases to an inactive state. The GAP activity of MYO9B is specific toward the Rho subfamily (particularly RhoA) with little to no activity toward other small GTPase subfamilies such as those typified by Rac1 or Cdc42 (Muller et al., 1997). MYO9B is particularly present in cellular protrusions where active actin polymerization occurs and is necessary for motility in multiple cell types (van den Boom et al., 2007; Hanley et al., 2010). In particular, MYO9B negatively regulates RhoA activity locally at sites of actin polymerization (Hemkemeyer et al., 2021), and its knockdown or deletion results in increased RhoA activity (Hanley et al., 2010; Chandhoke and Mooseker, 2012; McMichael et al., 2014).

We previously reported that loss of MYO9B in mice due to genetic knockout resulted in impaired growth, specifically during early puberty, with *Myo9b* ^*−/−*^ mice more severely affected than *Myo9b* ^*+/−*^ mice. Skeletal analysis of these animals at 4 and 12 weeks of age demonstrated *Myo9b* ^*−/−*^ femurs to be shorter in length than those from wild-type mice, with lower bone mineral density, trabecular bone volume and trabecular number, and decreased stiffness and cortical strength (McMichael et al., 2017; Kim et al., 2018). Heterozygous mice showed defects similar to those of homozygous knockouts at 4 weeks, but these were resolved by 12 weeks of age (McMichael et al., 2017; McMichael et al., 2017). Examination of bone-forming cells (osteoblasts) deficient in MYO9B revealed that they had defects in response to insulin-like growth factor 1 (IGF-1) but not to other growth factors due to impaired trafficking and activation of the IGF-1 receptor (IGF1R) (McMichael et al., 2017). Because of the importance of IGF-1 signaling in postnatal skeletal growth, these findings might fully explain the attenuated growth in these mice. However, we also found that osteoblasts completely lacking MYO9B demonstrated very poor adhesion to culture surfaces and contained fewer focal adhesions, suggesting that bone cells deficient in MYO9B might also be unable to properly respond to mechanical stresses (McMichael et al., 2017).

Osteocytes, which are terminally differentiated from osteoblasts, are by far the most abundant cells in bone. They perform a variety of functions in bone homeostasis that include matrix remodeling, modulation of osteoblast and osteoclast activities, and regulation of mineral metabolism (Robling and Bonewald, 2020). However, their unique function is as the primary sensors and transducers of mechanical signals in bone (Klein-Nulend et al., 1995). While their precursor osteoblasts are cuboidal cells that reside on bone surfaces, osteocytes are highly dendritic cells that are deeply embedded in bone in a fluid-filled network of voids called the lacuno-canalicular system (LCS). Within this fluid network, osteocytes are surrounded by a loose layer of collagen fibrils and other proteins called the pericellular matrix that may amplify strain at the cell membrane (You et al., 2001; Han et al., 2004; You et al., 2004). In addition, osteocyte dendrites come in direct contact with small, regularly spaced projections of the bone matrix termed canalicular projections or collagen hillocks (average of 130 ± 40 nm space between hillocks). Attachment of dendrites to these projections is through α_v_β_3_ integrin-based structures that possess features of classical focal adhesions but also associate with special channel proteins (Wang et al., 2007; McNamara et al., 2009; Cabahug-Zuckerman et al., 2018). These features, along with connexin-based (notably the Cx43 subunit) gap junctions in the connections between osteocyte processes contribute to making dendrites more sensitive sensors of mechanical stimuli than osteocyte cells bodies (Cheng et al., 2001; Cherian et al., 2005; Adachi et al., 2009; Burra et al., 2010; Thi et al., 2013). Osteocytes are exposed to a variety of mechanical stimuli, with fluid shear stress (FSS) playing a key role, particularly in cells cultured *in vitro* (Smalt et al., 1997a; Smalt et al., 1997b). Mechanical signaling via RhoA has been demonstrated to influence osteoblast differentiation and activity and has been identified in osteocytes (McBeath et al., 2004; Meyers et al., 2005; Arnsdorf et al., 2009; Hamamura et al., 2012; Wan et al., 2013; Nobis et al., 2017). As a regulator of RhoA activity, MYO9B is an excellent candidate for modulating responses to mechanical stimuli in bone. Therefore, studies were undertaken to determine how the lack of MYO9B influences mechanosensing in bone cells, including the bone-forming osteoblasts, and more specifically the primary mechanosensors of bone, the osteocytes.

## Materials and methods

### Cell culture and transfection/transduction

The murine osteoblast cell line MC3T3-E1 was cultured in GLUTAMAX αMEM (Gibco) containing 10% FBS (Atlanta Biologicals) and penicillin/streptomycin (Gibco) in a 5% CO_2_ incubator as previously described (McMichael et al., 2017). The mouse cell line MLO-Y4, an early differentiation stage osteocyte line [(Kato et al., 1997), a kind gift of Dr. Lynda Bonewald], was cultured in GLUTAMAX αMEM containing 5% fetal bovine serum (FBS, Atlanta Biologicals), 5% fetal calf serum (FCS, Hyclone) and penicillin/streptomycin (Gibco) in a 5% CO_2_ incubator on plates coated with monomeric collagen type I (Gibco). Cells were passaged twice weekly. Monomeric collagen coating was generated by adding to plates a solution of 150 μg/mL rat tail collagen (Gibco) in 0.02 M acetic acid for 1 h at 4°C, followed by brief rinses with phosphate-buffered saline (PBS) and air drying. For some experiments, polymerized (fibrillar) collagen I coating was generated by adding to plates a solution of 150 μg/mL rat tail collagen (Gibco) in PBS for 4 h at 37°C, followed by rinses with PBS and air drying. The monomeric and polymerized states of collagen in the above-mentioned conditions have been previously verified by us using AFM and collagen turbidity measurements (Agarwal et al., 2002; Mihai et al., 2006; Yeung et al., 2019).

Immunofluorescent labeling of MYO9B was performed by culturing MLO-Y4 cells on monomeric collagen-coated glass coverslips and fixing in 2% formaldehyde, permeabilizing, and labeling as previously described (McMichael et al., 2006). The rabbit anti-MYO9B primary antibody was from Proteintech and diluted 1:250 in blocking buffer composed of 20% FBS and 1% polyethylene glycol in PBS. Goat anti-rabbit Alexa Fluor-594 secondary antibody (1:1,000 in blocking buffer) and Alexa Fluor-488 phalloidin (1:1,000 in the same buffer as the secondary antibody) were purchased from Life Technologies.

For transient knockdown of MYO9B in MC3T3-E1 cells, non-targeting or targeting siRNAs were transfected using Ambion Silencer siRNAs (50 nM) with Lipofectamine RNAiMAX as previously described (McMichael et al., 2017). As assayed by qPCR using Taqman technology (Applied Biosystems), this method resulted in an average of 93% loss of MYO9B mRNA 1 day post-transfection, but only ∼70% loss of MYO9B protein on day 3 post-transfection, due to the inherent stability of myosin proteins (McMichael et al., 2017). For stable knockdown in MLO-Y4 cells, Mission shRNAs targeted to MYO9B in the lentiviral vector TRC2-PLKO-puro were obtained (TRCN0000362501, TRCN0000362502, Sigma-Aldrich). The vector containing a non-targeting shRNA was used for a control. Lentiviruses were generated using the Lenti-X system (Takara) and were titered using Takara GoStix to obtain GV units. Briefly, 5 × 10^4^ MLO-Y4 cells were seeded per 6-well in GLUTAMAX αMEM culture medium with serum (5% FBS, 5% FCS) but lacking antibiotics (day 0). The next day (day 1), 10,000–20,000 GV units of virus were added along with polybrene to a final concentration of 8 μg/mL. On day 2, old medium was removed and virus plus polybrene were added in fresh medium as on day 1. On day 3, the old medium was replaced with fresh culture medium containing serum and penicillin/streptomycin. On day 4, the cells were given fresh medium containing 15 μg/mL puromycin and were passaged in this medium for 2 weeks to ensure chromosomal integration of the viral genome. Cells were then cultured in standard medium without puromycin and were cloned by limiting dilution. Frozen stocks of each clone were prepared immediately after expansion. Upon thawing and passaging, relative *Myo9b* mRNA expression levels in knockdown cells were determined using TaqMan gene expression assays (Applied Biosystems) with 18 s rRNA as an endogenous reference. For Slit treatment of osteocytes, Slit1 and Slit3 were purchased from R&D Systems and were added individually to cells, 2 h after plating, at a concentration of 1 μg/mL followed by culture in normal growth medium overnight. Cells were then fixed in 2% formaldehyde, permeabilized, and stained for 45–60 min in crystal violet (0.2% in 2% ethanol). Photomicrographs were obtained and dendrite length was measured using ImageJ software.

### Administration of mechanical stress and cellular analysis

Uniaxial strain was applied to MC3T3-E1 cells using a Flexcell 4000 system. Briefly, cells were plated on 6-well Pronectin-coated UniFlex plates at 1 × 10^4^ cells per well, then were transfected with non-targeting or *Myo9b* siRNAs as described above. After 3 days, cells were subjected to 10% strain, 0.5 Hz for 4 h, then were immediately fixed in 2% paraformaldehyde and permeabilized. The region of uniaxial strain was excised from each well, and the membrane was placed on a microscope slide on a drop of water prior to staining with fluorescent phalloidin (Alexa Fluor-594, diluted 1:1,000) and mounting in ProLong Gold. Photomicrographs were obtained and stress fiber orientation was analyzed using the FibrilTool plug-in to ImageJ (Boudaoud et al., 2014). For mechanical stimulation of MLO-Y4, fluid shear stress was applied by orbital shaking (Fukada et al., 2017). Briefly, 0.5–1.0 × 10^5^ cells were plated in 6-wells in standard growth medium and cultured for 24 h. The medium was replaced with 1 mL fresh medium and cultured for 3 h more. The plates were placed on a rotating shaker platform at a fixed speed in a 5% CO_2_ incubator for 2 h, then were moved to a standard incubator overnight. Cells were fixed, permeabilized, and stained with crystal violet as above. Photos were taken of cells within 1 cm of the well’s edge and dendrite length was measured using ImageJ.

### Whole body vibration of mice

The Institutional Animal Care and Use Committee at The Ohio State University approved all protocols prior to use of animals in experiments. The university’s Animal Care and Use program assurance has been approved by the Office of Laboratory Animal Welfare (assurance #A3261-01). At 10 weeks of age, male wild-type C57Bl/6 and *Myo9b* ^*−/−*^ mice (a gift from Martin Bähler, Universität Münster) began an 8-week program of whole-body vibration (WBV). Mice were transferred from their housing cages to a subdivided acrylic box, which was placed on a vibration platform for 15 min. Mice receiving low-intensity WBV treatment were subjected to vibration of 0.3 × *g*, 90 Hz (Xie et al., 2006; Xie et al., 2008). Sham-treated mice were placed in the box on the platform but were not subjected to vibration. Vibration or sham treatment was performed 5 days a week for the 8-week period. Immediately after, mice were sacrificed and femurs were obtained for analysis.

### Bone analysis

A femur of each mouse was randomly selected and subjected to scanning by a 3D micro-CT scanner (SkyScan 1172-D, Kontich, Belgium) with voxel sizes at 20 × 20 × 20 μm^3^ under a consistent scanning condition of 70 kV, 141 μA, 0.4° rotation per projection, 8 frames averaged per projection, and 40 ms exposure time. A total of 29 femurs (10 WT WBV, 10 WT sham, 4 KO WBV, and 5 KO sham mice) were scanned and reconstructed. Bone and non-bone voxels in the image were segmented using a heuristic algorithm (Kim et al., 2012). Whole bone volume (BV) was obtained by multiplying the voxel size by the total number of bone voxels. Total volume (TV) was obtained including voxels of bone, pores, and marrow cavity. Then, a femoral bone fraction (BV/TV) was computed.

Tissue mineral density (TMD) is the mineral density of bone tissue, and unlike the more commonly used bone mineral density (BMD), does not include mineral measurements of bone marrow and pore spaces. Thus, TMD more precisely reflects mineral density of the bone tissue itself. TMD for each bone voxel was calibrated using a linear correlation curve between CT attenuation values and known densities of commercial phantoms provided by the micro-CT company. Total mineral content (TMC) was computed by multiplying the sum of TMD with the voxel size. BMD was the TMC in the TV. A mean of TMD was obtained by dividing the total sum of TMD by the total number of bone voxels. Lower and upper 5th percentile values of TMD distribution were also determined as Low_5_ and High_5_, respectively. Outer diameters of anterior-posterior axis and medial-lateral axis (D_AP_outer_ and D_ML_outer_) and perimeter were digitally measured at 55% of the femoral length from the head.

### Statistical analysis

Two-tailed t-tests were used to compare the parameters between cell groups and between sham and WBV groups of WT and KO mice. Significance was set at *p* < 0.05.

## Results

**FIGURE 1:**
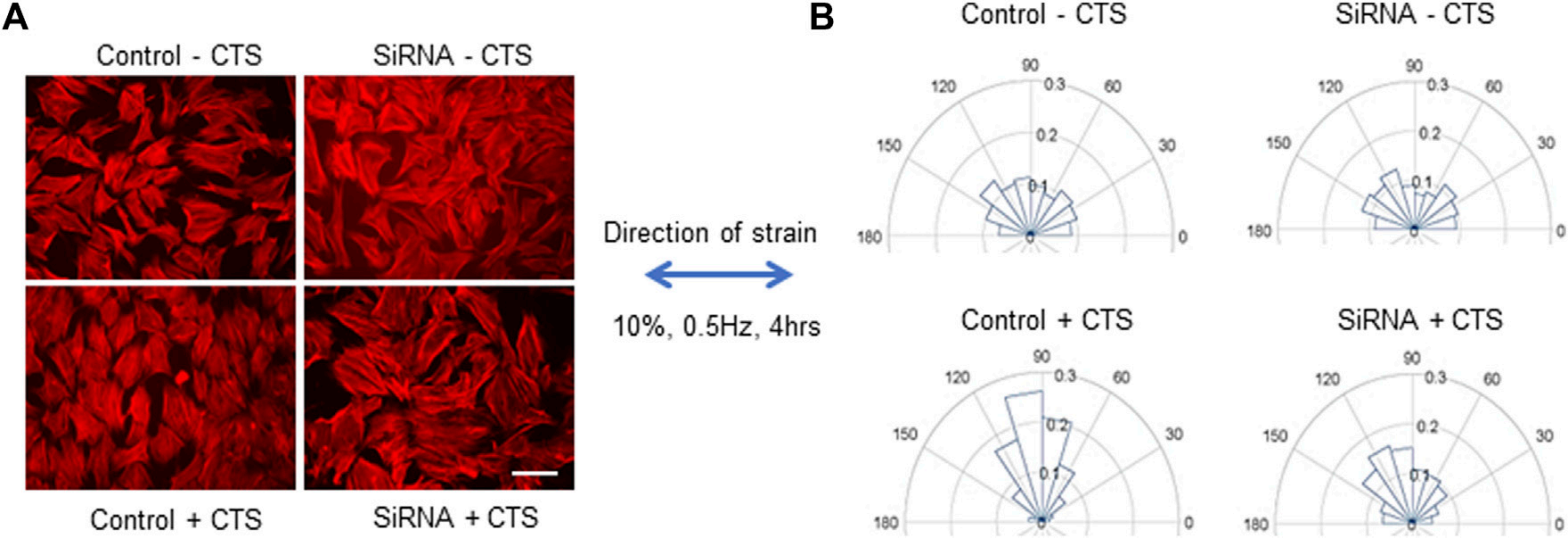
Responses of control or MYO9B-knockdown MC3T3-E1 cells to uniaxial cyclic tensile strain (CTS). Panel **(A)** shows fluorescent phalloidin labeling of cells treated with or without CTS. Scale bar = 20 μm. Panel **(B)** shows graphical orientations of the stress fibers in cells treated as in panel **(A)**. Data are compiled from 6 independent experiments with at least 750 cells per group.

Initial studies of the role of MYO9B in responses to mechanical stress were performed in the mouse osteoblastic cell line MC3T3-E1, in which expression of the myosin was knocked down by RNA interference, using methods previously described and resulting in ∼70% knockdown of MYO9B protein (McMichael et al., 2017). These cells were chosen for study rather than primary osteoblasts from *Myo9b* ^*−/−*^ mice, since complete knockout resulted in very poor adhesion of the cells to substrates. Both control- and siRNA-transfected cells were subjected to uniaxial cyclic tensile strain (CTS) of 10%, 0.5 Hz, for 4 h, then the cells were fixed and actin filaments were labeled with fluorescent phalloidin for analysis of stress fiber orientation. Under conditions of uniaxial CTS, normal cells have been established to re-orient from random directionality to a more uniform orientation perpendicular to the direction of strain, with stress fibers aligned in the direction of minimal substrate deformation (Buck, 1980; Wang et al., 2001; Faust et al., 2011). This reorientation was observed in the control MC3T3-E1 cells in Figures 1A, B. However, the reorientation was attenuated in cells in which MYO9B was knocked down (Figures 1A, B). These results indicate that the loss of MYO9B can negatively affect cellular responses to mechanical stress, likely due to excessive RhoA activity caused by this loss.

**FIGURE 2:**
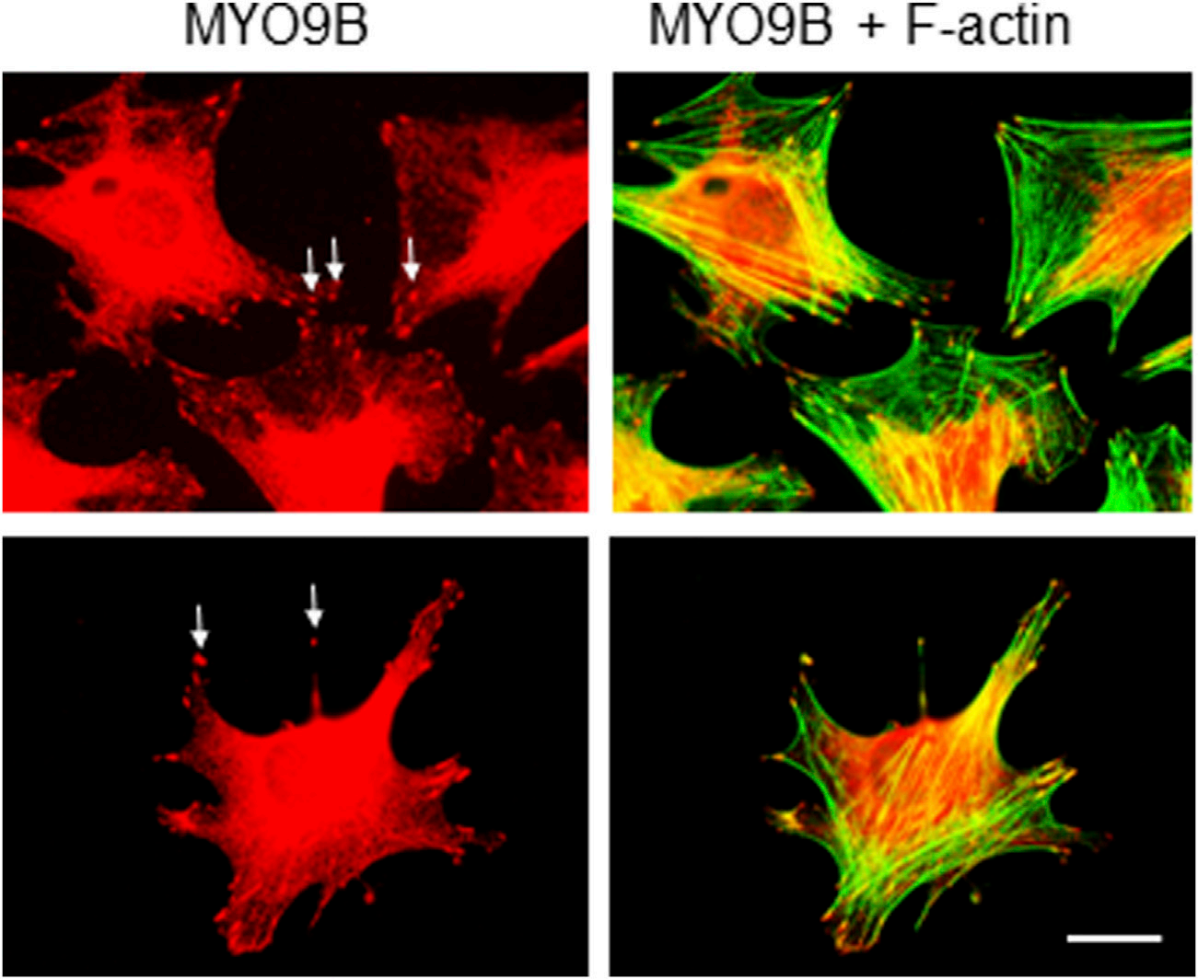
Immunofluorescent labeling of MLO-Y4 cells with antibody to MYO9B and fluorescent phalloidin. Arrows indicate MYO9B labeling at the tips of cell projections. Scale bar = 20 μm.

**FIGURE 3:**
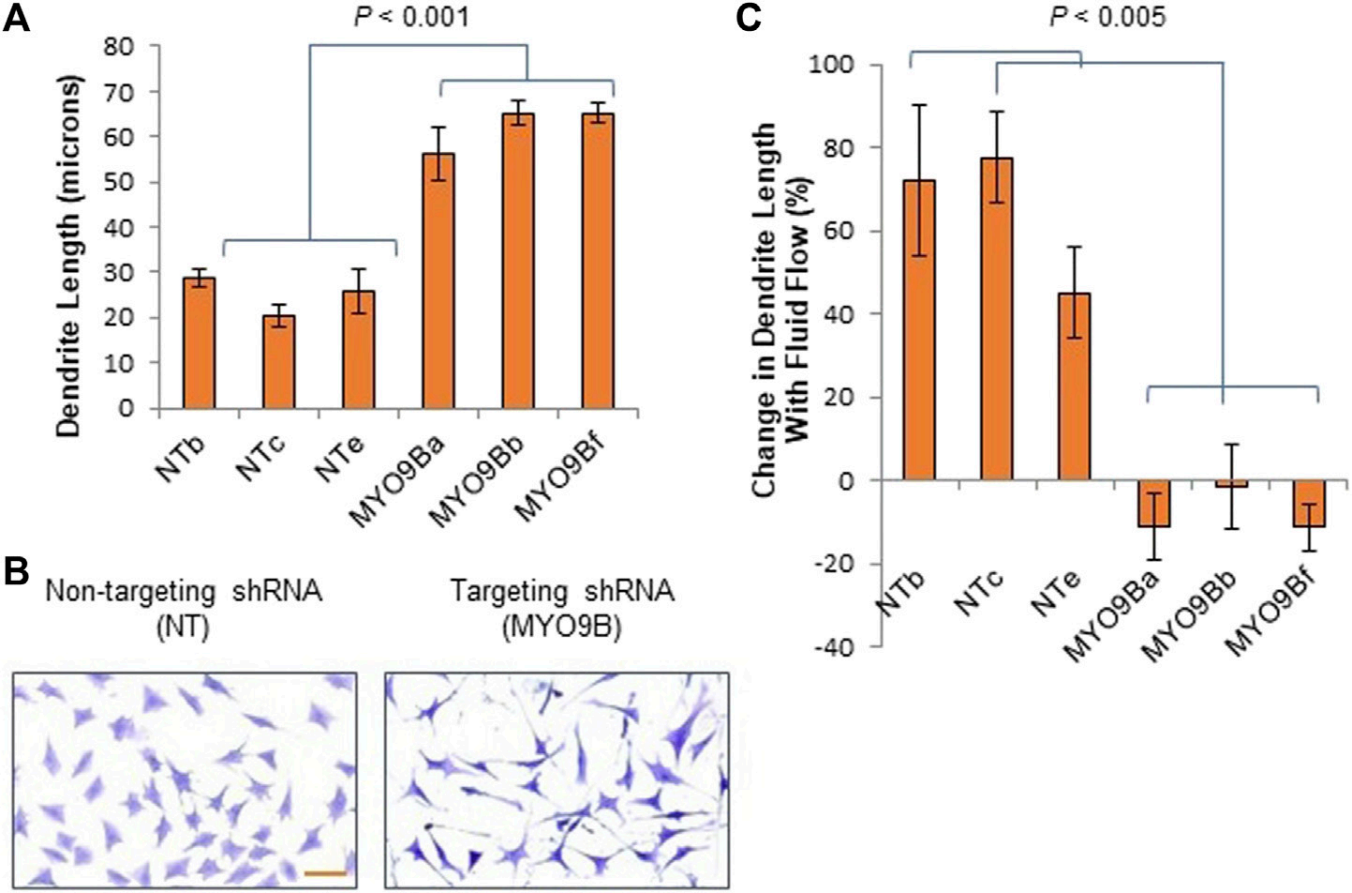
Dendrite responses of individual control (NT) or MYO9B-knockdown (MYO9B) MLO-Y4 cell clones to culture on monomeric collagen. Panel **(A)** illustrates quantification of dendrite length while **(B)** shows representative morphology of cells. Scale bar = 50 μm. Panel **(C)** shows changes in dendrite length following fluid shear stress. Each bar represents the average of 3 independent experiments with at least 100 cells measured in each experiment.

Because mechanosensing and responses by osteocytes play a key role in bone health, we set out to determine what roles MYO9B might play in osteocyte function. First, immunofluorescence microscopy was used to localize MYO9B in the early osteocyte cell line MLO-Y4. As shown in Figure 2, this protein was present in the ends of actin-based dendritic projections, as previously shown for other cell types (van den Boom et al., 2007; Hemkemeyer et al., 2021). Subsequently, MLO-Y4 cells were stably transduced with a lentiviral vector expressing shRNAs targeted to *Myo9b* mRNA or with a vector expressing a non-targeting (NT) control sequence. Initial inspection of resulting cells showed that those transduced with *Myo9b* shRNAs had longer dendrites than those transduced with the NT shRNA. Multiple clones of the resulting cells were isolated and expanded, and three knockdown clones plus three NT clones were randomly selected for detailed analysis. On average, the knockdown clones expressed 54.4% of the levels of *Myo9b* mRNA as the clones expressing the non-targeting sequence (range = 38.7–67.3%), as determined by TaqMan qPCR. As shown in Figures 3A, B, each individual clone in which MYO9B was knocked down had dendrites approximately 2- to 3-fold greater in length than control cells when grown on monomeric collagen I. To test whether these cells could respond to mechanical stimulation, both control and knockdown cells were subjected to fluid shear stress (FSS) as described in Materials and Methods. FSS was previously demonstrated to elongate dendrites in normal MLO-Y4 cells (Zhang et al., 2006). As shown in Figure 3C, control NT clones responded to the mechanical stress by increasing their dendrite length, while knockdown clones were unable to respond in this manner. These results suggest that dendrites of cells lacking MYO9B may have already reached a maximal length due to excessive RhoA activation and are unable to further elongate following stimulation from mechanical signals.

**FIGURE 4:**
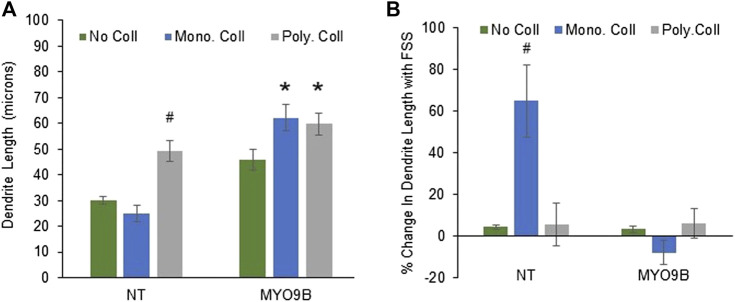
Dendrite responses of control (NT) or MYO9B-knockdown MLO-Y4 cells to plating on no collagen, monomeric type I collagen, or polymerized (fibrillar) type I collagen. Panel **(A)** shows dendrite length of untreated cells. Panel **(B)** shows changes in dendrite length following fluid shear stress (FSS). Symbols denote statistical differences from cells plated on no collagen; *: *p* < 0.01; #: *p* < 0.005. Each bar represents the average of 3 independent experiments with at least 100 cells measured in each experiment.

In the initial analysis just described, cells were plated on monomeric collagen, a standard method of culturing osteocytes that was shown to facilitate dendrite growth with FSS (Kato et al., 1997; Shah et al., 2016). However, bone is composed primarily of polymerized fibrils of collagen I; the monomeric form is primarily present during the initial stages of collagen I synthesis (Bou-Gharios et al., 2019). Therefore, to analyze the role of MYO9B in cells on a more physiologically relevant substrate, measurements of dendrites and fluid shear stress experiments were repeated in cells cultured on polymerized collagen I. In addition, these experiments were repeated in cells cultured on standard tissue culture plastic without collagen. Figure 4A demonstrates that NT cells plated on plastic or monomeric collagen I had similar length dendrites. However, plating on polymerized collagen I caused dendrite lengthening by approximately 100%. These results clearly indicate that monomeric and polymerized collagen I have differential effects on dendrites, with the polymerized form promoting greater length under baseline conditions. In contrast, MYO9B knockdown cell dendrites were elongated when plated on either monomeric or polymerized collagen. Cells in which MYO9B was knocked down had longer dendrites than NT cells on all substrates (on plastic, *p* < 0.005; on monomeric collagen, *p* < 0.001; on polymerized collagen *p* < 0.05).

When subjected to FSS, NT cells exhibited dendrite lengthening only on monomeric collagen I, as demonstrated in Figure 4B. Thus, the combination of plating on monomeric collagen I plus FSS promoted dendrite length, demonstrating the interaction between the substrate and mechanical stress. No lengthening of dendrites in MYO9B knockdown cells was observed in the presence of FSS on any substrate. These results show that MYO9B-deficient cells, which already possess long dendrites, are unable to lengthen their dendrites further in response to FSS.

**FIGURE 5:**
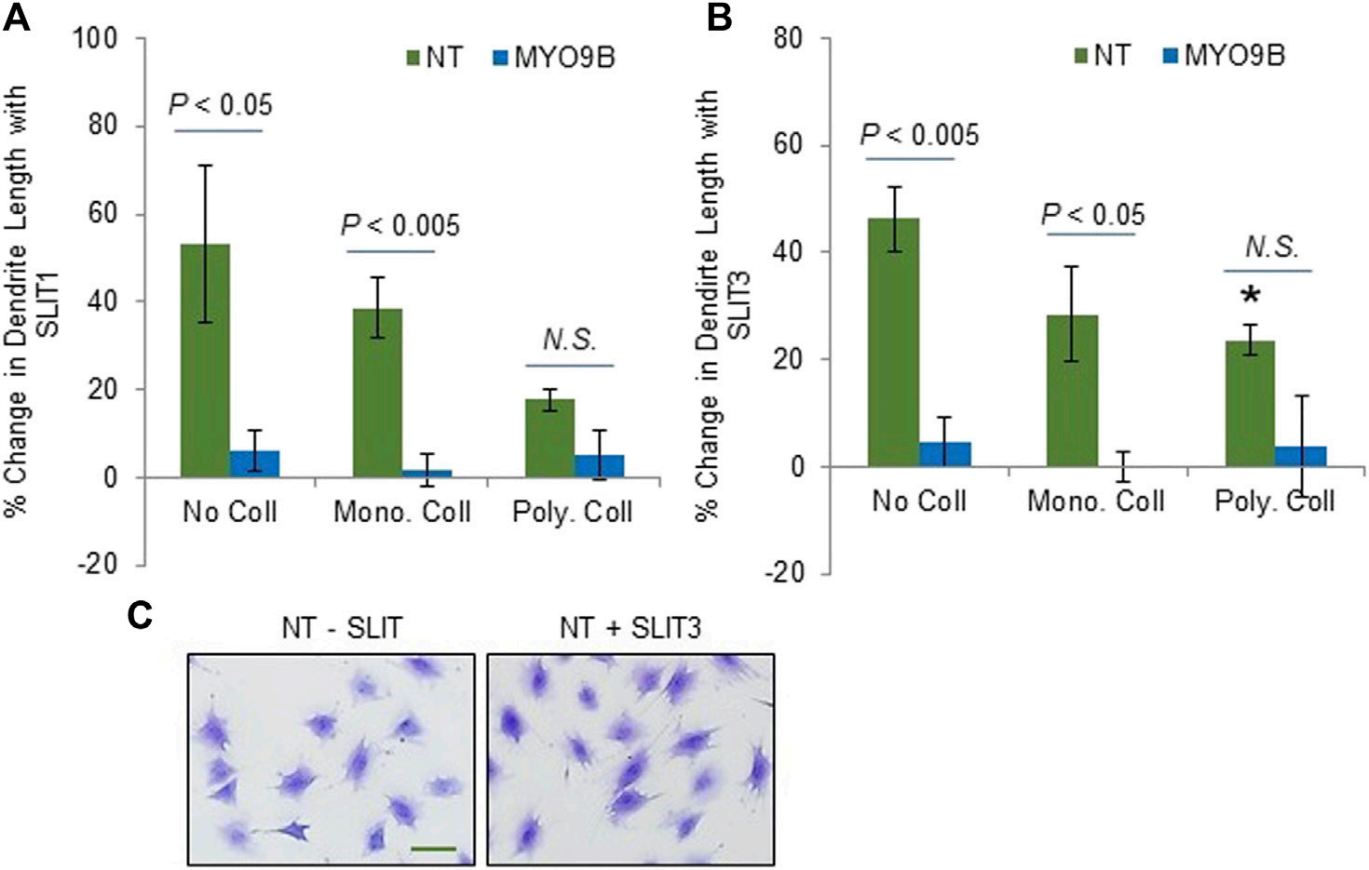
Dendrite responses of control (NT) or MYO9B-knockdown MLO-Y4 cells to treatment with **(A)** Slit1 or **(B)** Slit3. Cells were plated on no collagen or monomeric or polymerized type I collagen. The asterisk denotes statistical difference from cells plated on no collagen. *N.S.* = not significant. Each bar represents the average of 3 independent experiments with at least 100 cells measured per experiment. Panel **(C)** shows representative NT cells on monomeric collagen I with or without SLIT3 treatment. Scale bar = 20 μm.

Slit ligands are soluble factors that bind to Roundabout (Robo) receptors and are well established as neuronal guidance molecules (Blockus and Chedotal, 2016). Slit-Robo signaling is increasingly explored in other processes such as tumor development and angiogenesis and recently, bone metabolism (Jiang et al., 2022). Slit2 and Slit3 both were shown to be expressed by osteoblasts and osteoclasts and to affect their differentiation and activity, though the bone-specific source of Slit3 is still under question (Sun et al., 2009; Kim et al., 2018; Park et al., 2019; Li et al., 2020). Further, multiple Robo receptors have been identified in bone cells (Sun et al., 2009; Kim et al., 2018; Park et al., 2019). Interestingly, MYO9B was shown to physically interact with the intracellular domain of Robo1 in lung cancer cells, leading to MYO9B inhibition and RhoA activation upon Slit addition. MYO9B was also demonstrated to be required for Slit-induced RhoA activation (Kong et al., 2015). To determine whether components of Slit-Robo signaling were present in MLO-Y4 cells, RT-PCR primers were designed specifically to amplify Robo 1–4 and Slit 1–3. Strong amplification products were detected for Robo1 and Slit 2 (data not shown). Then, to determine whether Slit treatment would alter dendrite length in osteocytes with intact MYO9B levels, NT cells were treated overnight with Slit1 or Slit3. As shown in Figures 5A–C, treatment of NT cells with either soluble factor increased dendrite length. However, this effect was less prominent in cells on polymerized collagen (which have a baseline of long dendrites) relative to cells on monomeric collagen or no protein substrate. In contrast, MYO9B knockdown cells were not further affected by treatment with Slit factors (Figures 5A–C). These results demonstrate that Slit-Robo signaling is present in osteocytic cells and can promote dendrite elongation, but that MYO9B deficiency prevents this response. However, this experiment does not determine whether MYO9B knockdown prevents Slit-mediated dendrite elongation due to lack of MYO9B-Robo interactions or whether the dendrites are simply at a maximal length due to excessive RhoA signaling and cannot be further extended. More experiments are needed to differentiate between these possibilities. Nonetheless, the responses of NT and MYO9B cells to varying collagen substrates and FSS or Slit treatment are summarized in Table 1.

**TABLE 1:** Dendrite responses to exogenous signals in cultured MLO-Y4 cells. Low MYO9B levels or plating on polymerized collagen I results in cells with lengthened dendrites and a diminished ability to further lengthen dendrites upon stimulation with fluid shear stress (FSS) or Slit signaling. Culture of normal cells in the absence of collagen also inhibits dendrite lengthening from FSS but does not inhibit the response to Slit signaling.

	Collagen I substrate	Base dendrite length	Dendrite response to FSS	Dendrite response to slit signaling
	*None*	Moderate	---	+
Normal MYO9B	*Monomeric*	Moderate	+	+
	*Polymerized*	Long	---	+/−
	*None*	Long	---	---
Low MYO9B	*Monomeric*	Long	---	---
	*Polymerized*	Long	---	---

**FIGURE 6:**
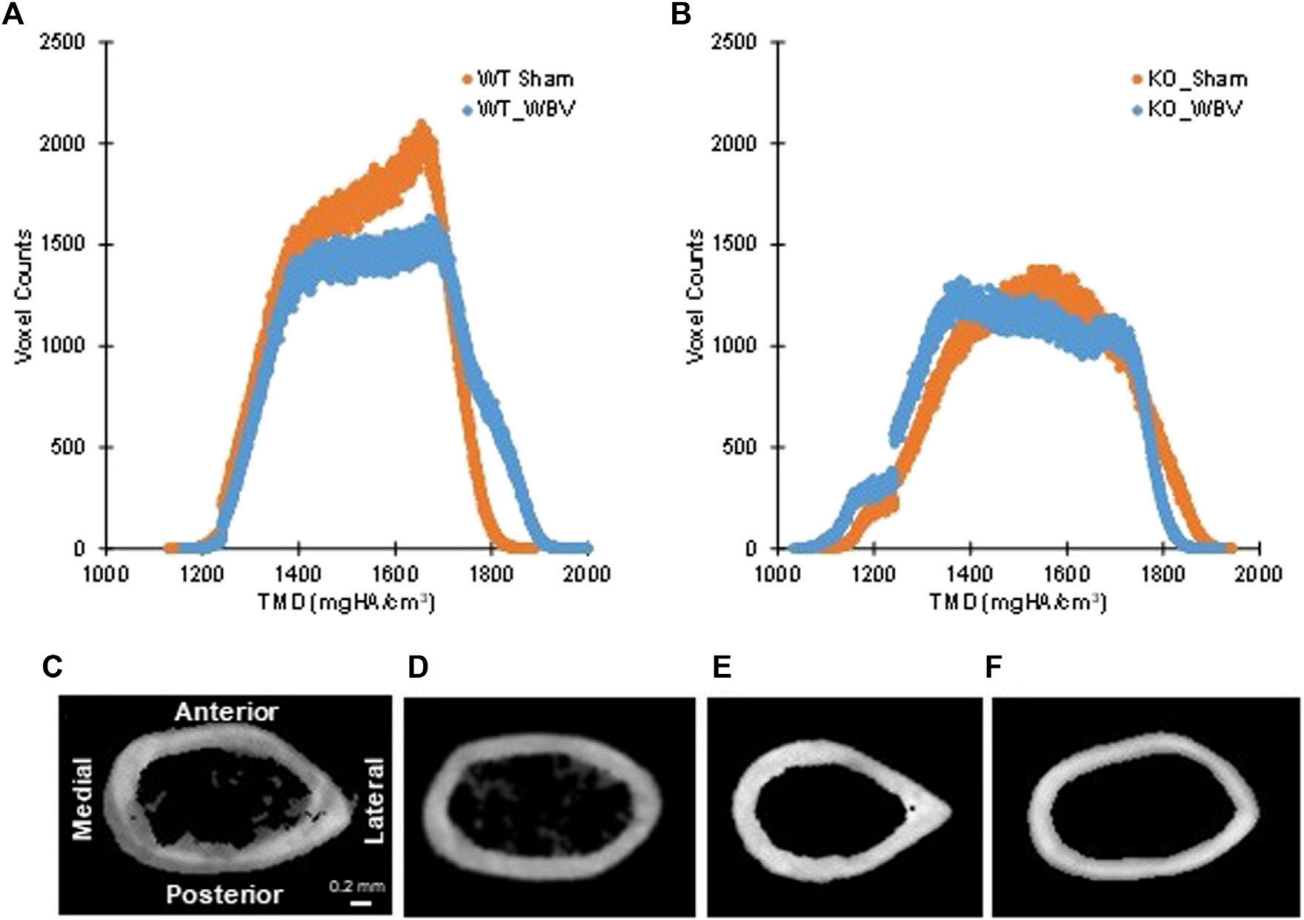
Panels **(A,B)**: Tissue mineral density (TMD) parameters in individual histograms of typical whole bone for **(A)** WT sham and WBV groups and **(B)** KO sham and WBV groups. As previously reported in 12-week-old mice, the knockout group had significantly less mineralized bone tissue (i.e. voxel count) than the wild-type group (see ref. 6). Changes in tissue mineral density with WBV can be observed by shifts of the curve leftward or rightward. Panels **(C–F)**: Typical cross-sectioned cortical regions at 55% of the femoral length from the head for **(C)** WT sham, **(D)** WT WBV, **(E)** KO sham, and **(F)** KO WBV groups. *N* = 4–10 per group.

These findings clearly show that MYO9B plays a role in osteocyte dendrite growth. The absence of MYO9B results in osteocytes with elongated dendrites that are unable to respond to the ECM environment or mechanical or Slit stimulation with further dendrite lengthening. At the same time, it is clear that the state of collagen I underlying these cells can also affect these responses. Therefore, to determine whether a lack of MYO9B plays a role in sensing or transducing mechanical signals *in vivo*, male wild-type or *Myo9b* ^*−/−*^ mice underwent whole body vibration (WBV), a procedure known to stimulate bone growth in humans and animals (Thompson et al., 2014). Young mice (10 weeks of age) were subjected to 15 min of WBV (0.3 × *g*, 90 Hz), 5 days/week for 8 weeks or sham treatment. From analysis of femurs, we found that WBV induced an increase in overall tissue mineral density (TMD; Mean values, Table 2) as well as increased mineral in both newly formed and mature bone (Low_5_ and High_5_ values, Table 2). These changes can be visualized as shifting of the TMD curve to the right in Figure 6A. In contrast, femurs from *Myo9b* ^*−/−*^ mice showed none of the same responses to WBV as wild-type mice. Instead, WBV in these mice resulted in decreased bone mineral density (BMD, Table 2), expansion of the medullary space with an accompanying widening of the femur diameter (cortical bone diameters, Table 2 and Figures 6C–F), and an overall loss of bone volume (BV/TV, Table 2). Further, WBV produced a leftward shift in the TMD curve (Figure 6B). These results demonstrate MYO9B’s role in responses to mechanical stresses *in vivo* and show that while wild-type mice responded to WBV with increased tissue mineral density, *Myo9b* ^*−/−*^ mice responded to the same treatment with bone loss through different parameters.

**TABLE 2:** Comparison of measured parameters between sham and WBV treatments (mean ± standard deviation) in male femur of WT and KO groups. Statistically significant differences between Sham and WBV groups (*p* < 0.05) are highlighted in bold.

Parameters		WT			KO		
		Sham	WBV	*p*-value	Sham	WBV	*p*-value
Volumetric	BV (mm^3^)	26.34	23.87	0.90	21.68	24.29	0.12
		±1.94	±3.15		±0.97	±3.17	
	TV (mm^3^)	44.56	41.75	0.83	**35.56**	**41.4**	**0.05**
		±2.16	±4.45		**±1.9**	**±5.19**	
	BV/TV	0.59	0.57	0.96	**0.61**	**0.59**	**0.01**
		±0.02	±0.03		**±0.01**	**±0.01**	
Mineral Density	TMC (mgHA)	40.26	37.39	0.86	33.12	36.63	0.18
		±3.16	±5.63		±1.44	±5.13	
	BMD (mgHA/cm^3^)	902.61	893.35	0.83	**931.97**	**883.64**	**0.01**
		±33.49	±70.14		**±19.57**	**±18.34**	
	**Mean (mgHA/cm** ^**3**^ **)**	**1,528.28**	**1,563.31**	**0.03**	1,527.68	1,506.93	0.11
		**±14.41**	**±42.24**		±16.67	±17.62	
	SD (mgHA/cm^3^)	137.58	153.55	0.06	158.14	155.75	0.52
		±6.13	±23.45		±5.45	±4.87	
	**Low**_**5**_ **(mgHA/cm** ^**3**^ **)**	**1,306.05**	**1,320.11**	**0.02**	**1,266.77**	**1,249.04**	**0.04**
		**±6.36**	**±15.26**		**±9.32**	**±12.3**	
	**High**_**5**_ **(mgHA/cm** ^**3**^ **)**	**1745.95**	**1807.14**	**0.04**	1767.27	1740.92	0.09
		**±20.08**	**±80.75**		±22.89	±15.41	
Cortical bone diameters	D_AP_outer_ (mm)	1.33	1.29	0.2	**1.13**	**1.26**	**0.01**
		±0.07	±0.05		**±0.02**	**±0.08**	
	D_AP_inner_ (mm)	0.91	0.91	0.84	**0.76**	**0.88**	**0.02**
		±0.06	±0.05		**±0.05**	**±0.08**	
	Perimeter55 (mm)	5.83	5.73	0.29	**5.06**	**5.66**	**0.03**
		±0.19	±0.21		**±0.15**	**±0.17**	

## Discussion

Bone metabolism relies on a complex interplay of activities by bone forming cells, bone degrading cells, and mechanosensing cells. RhoA, the regulatory target inhibited by MYO9B, has been demonstrated to play key roles in bone cell functions, including osteoclast motility and resorption, osteoblast differentiation and bone formation, and osteocyte mechanosensing (Nobis et al., 2017; Strzelecka-Kiliszek et al., 2017). As a regulator of RhoA, it is unsurprising that a loss of MYO9B activity would negatively impact bone health. Our earliest studies of MYO9B’s role in bone showed that cultured osteoclasts with diminished MYO9B expression were attenuated in their ability to resorb bone (McMichael et al., 2014); however, bone resorption was not significantly impaired in *Myo9b* ^*−/−*^ mice. Instead, *Myo9b* ^*−/−*^ mice had smaller, weaker bones due to diminished bone formation (McMichael et al., 2017; McMichael et al., 2017). Although primary osteoblasts from *Myo9b* ^*−/−*^ mice could not be cultured due to their inability to adhere to substrate, we found that the osteoblast cell line MC3T3-E1, when treated with *Myo9b*-targeting siRNAs, had impaired responses to IGF-1 but not other assayed growth factors. This finding could certainly explain the impaired growth of *Myo9b* ^*−/−*^ mice, which demonstrated normal growth rates except for the period from 3 weeks to 8 weeks of age, when IGF-1-mediated growth is at its highest levels. The interactions between MYO9B and IGF-1 signaling are unclear, although further studies demonstrated that MYO9B-deficient cells showed altered trafficking, expression, and activation of the IGF-1 receptor IGF1R ((McMichael et al., 2017) and manuscript in preparation). However, given MYO9B’s effects on actin cytoskeletal organization and the known role of RhoA in mechanosignaling, this study highlights how alterations in RhoA activity might affect osteocyte function and mechanosignaling in bone.

To first demonstrate how MYO9B might regulate responses to mechanical stress, we subjected the mouse osteoblast line MC3T3-E1 with normal or deficient levels of MYO9B to uniaxial strain and tested for the cells’ ability to re-orient in response to this strain. Unlike normal control MC3T3-E1, cells deficient in MYO9B could not re-orient perpendicular to the direction of strain. We previously found that osteoblasts lacking MYO9B demonstrated poor adhesion to substrate and expressed fewer focal adhesions than control cells (McMichael et al., 2017). These attributes could readily explain the MYO9B-deficient cellular responses to mechanical stress. Subsequently, we found that diminished levels of MYO9B had clear effects on the ability of osteocytic dendrites to respond to signals that promote growth of these processes (Table 1). The loss of MYO9B alone resulted in cells with longer dendrites, suggesting that MYO9B’s role in osteocytes is to act as a brake on dendrite growth. By maintaining osteocyte dendrites at a moderate length, MYO9B allows the cells to respond to exogenous signals such as fluid shear stress and Slit-Robo signaling with dendrite elongation. In the absence of MYO9B, it is expected that dendritic RhoA is excessively activated, leaving little opportunity for RhoA-mediated responses to these exogenous signals. That said, we found that the state of the collagen that interacts with osteocytes played a marked role in mechanically-induced dendrite lengthening in non-shRNA-treated cells. The lack of collagen I resulted in osteocytes with moderate-length dendrites that could be extended with Slit treatment but not with fluid shear stress. Monomeric collagen I substrate produced cells with moderate length dendrites and facilitated dendrite lengthening from both fluid shear stress and Slit signaling. In contrast, plating cells on polymerized collagen I resulted in osteocytes with long dendrites, an inability to respond to fluid shear stress, and only a modest ability to respond to Slit signaling. Although these findings result from culturing osteocytic cells on an artificial 2-dimensional substrate, they do have implications for cells in their native 3-dimensional bone environments. As briefly described above, osteocytes exist *in vivo* in a fluid-filled lacuno-canalicular system. However, their dendrites do come in contact with collagen fibrils from canalicular projections (Wang et al., 2007; McNamara et al., 2009). How these sporadic attachments influence dendritic responses to exogenous signals is unclear; further, the formation and precise composition of these projections is unknown, leaving numerous questions about their interactions with dendrite formation and responsiveness. A particularly interesting finding in this study is the differences in responses when cells were plated on monomeric *versus* polymerized collagen. Cells on the monomeric form showed greater dendritic sensitivity to either fluid shear stress or Slit signaling. If this is also true *in vivo*, it is easy to imagine a scenario in which nascent osteocytes becoming surrounded by newly forming collagen I matrix (which contains monomeric collagen) might show greater dendritic growth in response to exogenous signals than mature osteocytes in a fully formed bone matrix.

Although many aspects of osteocyte mechanosensing are well-studied, our knowledge of the mechanisms involved in dendrite growth is still limited. However, it is established that elongation of these processes is promoted by RhoA activity and mechanical stimulation. The glycoprotein E11 (also known as Gp38 or podoplanin) is an early marker of the osteoblast-to-osteocyte transition and is highly expressed in the dendrites of these developing cells (Zhang et al., 2006). E11 is present in dendrites of both early osteocytes that reside in maturing bone (osteoid) and in differentiated osteocytes that are more deeply embedded in mineralized bone. SiRNA-mediated knockdown of E11 in cultured cells attenuated dendrite growth in response to fluid shear stress (Zhang et al., 2006). Further, increased expression of E11 in an early osteocyte cell line by inhibition of its proteolytic degradation resulted in concurrent activation of RhoA and elongation of dendritic processes (Staines et al., 2016). This study confirms earlier work showing that E11 activates RhoA activity in other cell types (Martin-Villar et al., 2006) and demonstrates the role of RhoA in E11-mediated osteocyte dendrite growth. Thus, it is highly likely that MYO9B, by inhibiting RhoA, works in balance with E11 to modulate osteocytic process formation under a variety of physiological conditions.

Because cells with low levels of MYO9B were deficient in their responses to fluid shear stress and Slit signaling *in vitro*, we tested whether the bones of *Myo9b* ^*−/−*^ mice could respond to mechanical stimulation *in vivo* via whole-body vibration. We found that this protocol increased tissue mineral density in the femurs of wild-type mice, but the femurs of *Myo9b* ^*−/−*^ mice showed decreased bone mineral density and TMD parameters primarily due to expansion of the medullary space. These data indicate that both mouse strains could respond to mechanical stimuli, though wild-type mice responded with bone formation, suggesting net increased osteoblastic activity. In contrast, *Myo9b* ^*−/−*^ mice responded with bone loss, suggesting net increased osteoclastic activity. The precise reasons for these differences are as yet unclear. Low magnitude, high frequency vibrations of MLO-Y4 cells (similar to the conditions used on mice in these studies) were shown to strongly reduce osteocytic production of osteoclast differentiation factors (RANKL, PGE_2_), potentially leading to WBV-inhibited osteoclast formation in wild-type animals (Lau et al., 2010). It is possible that in the *Myo9b* ^*−/−*^ mice, this osteoclast inhibition does not occur, leading to eventual bone loss. Another possibility is that bone loss may be due to decreased numbers of mature osteogenic cells in *Myo9b* ^*−/−*^ mice. We were unable to test differentiation of osteogenic precursors from these mice in culture due to their inability to remain attached to culture substrates long-term (McMichael et al., 2017). However, male *Myo9b* ^*−/−*^ mice express similar numbers of osteoblasts as male WT mice (McMichael et al., 2017), and although osteocyte numbers were not quantified in these animals, no evidence of fewer osteocytes in the knockout mice was noted. More detailed studies are required to determine whether *Myo9b* ^*−/−*^ osteocytes might adopt different morphologies in dendrite length and number than wild-type osteocytes, or whether connectivities between dendrites and neighboring bone cells might be altered. Alternately, the defects in *Myo9b* ^*−/−*^ osteocytes might be functional, rather than structural in nature. For example, the altered RhoA activity in these cells could give rise to changes in downstream pathways such as Rho kinase signaling pathways. Alternately, it is possible that the osteocytes in these mice are functional, and the previously described defects in osteoblast responses to IGF-1 may be the foundation of the altered response to mechanical stimuli in *Myo9b* ^*−/−*^ mice. Indeed, previous work has indicated that intact IGF-1 signaling is required for bone mechanosensitivity (Lau et al., 2013). IGF-1 expression by osteocytes is strongly induced by mechanical stimulation, and it is proposed that this growth factor acts directly on neighboring osteoblasts to mediate the osteogenic effects of this stimulation (Lean et al., 1995; Kawata and Mikuni-Takagaki, 1998; Reijnders et al., 2007; Lau et al., 2013). Because osteoblasts deficient in MYO9B have diminished responsiveness to IGF-1 signaling, they may simply become unresponsive to the effects of mechanical stimulation, leading to attenuated bone formation and altered osteoclast signaling. Further studies are required to distinguish among these myriad possibilities.

In summary, as a regulator of RhoA signaling, MYO9B plays a crucial role in mediating responses to mechanical stimuli in bone cells. *In vitro*, deficiency of MYO9B leads to diminished responses to substrate deformation by osteoblasts. It also leads to enhanced dendrite growth in osteocytes and an inability of the osteocytes to respond to dendrite elongation signals. *In vivo*, knockout of MYO9B leads to a lack of mechanically-induced bone formation and production of a net bone loss. While more detailed analyses of these systems are necessary for full understanding of how MYO9B influences mechanosignaling in bone, these studies further highlight how regulation of RhoA and the actin cytoskeleton influence skeletal health.

## Data Availability

The raw data supporting the conclusion of this article will be made available by the authors, without undue reservation.
